# Draft Genome Sequences of *Anaplasma phagocytophilum*, *A. marginale*, and *A. ovis* Isolates from Different Hosts

**DOI:** 10.1128/genomeA.01503-17

**Published:** 2018-02-01

**Authors:** Sandra Diaz-Sanchez, Angélica Hernández-Jarguín, Isabel G. Fernández de Mera, Pilar Alberdi, Erich Zweygarth, Christian Gortazar, José de la Fuente

**Affiliations:** a SaBio, Instituto de Investigación en Recursos Cinegéticos IREC (CSIC-UCLM-JCCM), Ciudad Real, Spain; b Institute for Parasitology and Tropical Veterinary Medicine, Free University of Berlin, Berlin, Germany; c Department of Veterinary Tropical Diseases, Faculty of Veterinary Science, University of Pretoria, Onderstepoort, South Africa; d Department of Veterinary Pathobiology, Center for Veterinary Health Sciences, Oklahoma State University, Stillwater, Oklahoma, USA

## Abstract

Here, we report the draft genome sequences of isolates of *Anaplasma phagocytophilum*, *Anaplasma marginale*, and *Anaplasma ovis*. The genomes of *A. phagocytophilum* (human), *A. marginale* (cattle), and *A. ovis* (goat) isolates from the United States were sequenced and characterized. This is the first report of an *A. ovis* genome sequence.

## GENOME ANNOUNCEMENT

The genus *Anaplasma* (*Rickettsiales*: *Anaplasmataceae*) comprises obligatory intracellular Gram-negative bacteria that are mainly transmitted by ticks, so far including seven species, *Anaplasma phagocytophilum*, *A. marginale*, *A. ovis*, *A. bovis*, *A. centrale*, *A. platys*, and *A. capra* (1, 2). These pathogens cause different forms of anaplasmosis in humans and domestic and wild animals worldwide (3). Recently, several studies have reported genome sequence information for *Anaplasma* spp. to advance the identification of candidate protective antigens and knowledge of genetic diversity, host tropism, virulence, and tick transmissibility of these pathogens (4–9). Currently, sequence information is available for 29 and 14 genomes for *A. phagocytophilum* and *A. marginale*, respectively, and 1 genome for *A. centrale*. However, genome sequence information is not available for other *Anaplasma* spp. such as *A. ovis*, which was included in this study.

Here, we report the draft genome sequences of the strains *A. phagocytophilum* NY18 (10), *A. marginale* Oklahoma-2 (11, 12), and *A. ovis* Idaho (12, 13), which were isolated in the United States from human, cow, and goat, respectively. The isolates were grown in cultured *Ixodes scapularis* IDE8 or ISE6 cells as previously described (11), and chromosomal DNA samples were obtained by using the DNeasy blood and tissue and MinElute PCR purification kits (Qiagen, Valencia, CA, USA) according to the manufacturer’s protocols. Genomic DNA was subjected to fragmentation using Agencourt AMPure XP (Beckman Coulter, Brea, CA, USA) to obtain DNA fragments of an average final size of about 500 bp. Samples were then used to prepare sequencing-amenable TruSeq libraries (NEB-Next, New England Biolabs, Ipswich, MA, USA). The libraries were quantitated with quantitative PCR (qPCR), and DNA was then denatured and equilibrated so that a final library concentration of 10 pM was loaded onto a MiSeq version 3 flow cell (Illumina, San Diego, CA, USA) and sequenced using a 2 × 250 paired-end sequencing protocol with >74% of the bases showing a Q30 factor of >30. Genome assembly and analysis were conducted by CD Genomics (Shirley, NY, USA). After processing with FastQC (https://www.bioinformatics.babraham.ac.uk/projects/fastqc/) for quality control, high-quality reads were assembled using the short oligonucleotide analysis package SOAPdenovo2 (version 2.04) (http://soap.genomics.org.cn/soapdenovo.html). The assembled results were optimized according to the paired-end and overlap relations of the reads by using GapCloser (version 1.12) (http://soap.genomics.org.cn/soapdenovo.html) to repair the results of the assembly hole and remove the redundant sequences from the final assembly. The protein-coding genes were predicted using Glimmer 3.02 (https://ccb.jhu.edu/software/glimmer/), and tRNAscan-SE (http://lowelab.ucsc.edu/tRNAscan-SE/) and RNAmmer (http://www.cbs.dtu.dk/services/RNAmmer/) were used to identify tRNA and rRNA, respectively. The genome sequences were also uploaded into Rapid Annotations using Subsystems Technology (RAST) (14) to check the annotated sequences. The assembled genomes were mapped to reference genomes (*Anaplasma phagocytophilum* strain HZ [GenBank accession number NC_007797] and *Anaplasma marginale* strain Florida [NC_012026]) using SOAPaligner (version 2.21) (http://soap.genomics.org.cn/soapaligner.html).

The sequenced genomes consisted of 1,210 (*A. phagocytophilum* NY18), 1,033 (*A. marginale* Oklahoma-2), and 1,034 (*A. ovis* Idaho) genes. The availability of these genome sequences from field *Anaplasma* isolates will allow comparative analysis to other *Anaplasma* species to expand the study of the evolution and host specificity of these pathogens and to find correlates with phenotypic variation with implications for anaplasmosis disease risk assessment and control.

### Accession number(s).

The genome sequences were deposited in GenBank under accession numbers PKOG00000000 (*A. phagocytophilum* NY18), PKOF00000000 (*A. marginale* Oklahoma-2), and PKOE00000000 (*A. ovis* Idaho).
